# Facteurs de risque de mortalité chez les nouveaux-nés transférés au service de néonatologie de l'Hôpital Jason Sendwe de Lubumbashi, République Démocratique du Congo

**DOI:** 10.11604/pamj.2014.19.169.4018

**Published:** 2014-10-17

**Authors:** Tina Katamea, Olivier Mukuku, Lewis Kamona, Kethye Mukelenge, Otikeke Mbula, Lambert Baledi, Emile Ntambwe, Augustin Mulangu Mutombo, Stanis Okitotsho Wembonyama, Oscar Numbi Luboya

**Affiliations:** 1 Faculté de Médecine, Université de Lubumbashi, Lubumbashi, République Démocratique du Congo; 2 Centre Médical Light, Lubumbashi, Lubumbashi, République Démocratique du Congo; 3 Ecole de Santé Publique, Université de Lubumbashi, Lubumbashi, République Démocratique du Congo

**Keywords:** Transfert extrahospitalier, nouveau-né, facteurs de risque, Lubumbashi, Extra-hospital transfer, newborn, risk factors, Lubumbashi

## Abstract

**Introduction:**

La mortalité néonatale reste préoccupante dans notre milieu et plusieurs facteurs dont ceux liés à l'organisation du transfert de nouveau-nés au niveau des structures de prise en charge y contribuent. Les objectifs de notre étude sont de déterminer la fréquence du transfert néonatal extrahospitalier à l'unité de néonatologie et identifier les facteurs de risque de mortalité dans notre milieu.

**Méthodes:**

Il s'agit d'une étude prospective et analytique menée sur une période de douze mois allant du 1er Janvier 2012 au 31 décembre 2012 ayant ciblé tous les nouveau-nés transférés des maternités extérieurs, traités et suivis dans le service de néonatologie de l'hôpital Sendwe. Les caractéristiques maternelles (âge, parité, état-civil, profession, niveau d’étude, antécédents médicaux et toxicologiques) et perpartales (âge gestationnel, état de la poche des eaux, voie d'accouchement) et néonataux (âge lors du transfert, sexe, poids de naissance, moyen de transfert, motif de transfert et évolution (guérison ou décès)) ont été étudiés. La saisie et l'analyse des données ont été faites sur le logiciel Epi Info 2011 (version 7.0.8.3) et le seuil de signification de 5%.

**Résultats:**

La fréquence du transfert néonatal extrahospitalier est de 12,9%. La mortalité était significativement liée à la profession (vendeuse ou travailleuse) de la mère (OR = 7,43 (1,38-39,97)), au niveau d’étude élevé (OR = 4,22 (1,18-15,10)), à l’âge gestationnel <37 SA (OR=3,21 (1,46-7,06)), à l’accouchement par voie basse de manière dystocique (OR=6,50 (1,54-27,38)), au sexe masculin du nouveau-né (OR=2,43 (1,08-5,46)), au poids de naissance <1500 grammes (OR=15,3 (5,73-40,78)) et à la prématurité comme motif de transfert (OR=5,56 (1,47-20,98)).

**Conclusion:**

Le bilan de la mortalité néonatale est lourd dans les pays en développement où les nouveau-nés continuent de mourir pour des causes souvent évitables. La réduction de la morbidité et la mortalité néonatales passe par une amélioration du système de référence des nouveau-nés dans notre milieu.

## Introduction

Le quatrième Objectif du Millénaire pour le Développement (OMD), visant à réduire la mortalité infantile de 2/3 d'ici 2015, passe par l'amélioration de la qualité des soins obstétricaux et néonataux permettant ainsi de réduire la mortalité néonatale. Environ 4 millions de décès néonataux sont enregistrés chaque année dans le monde entier, et trois quart d'entre eux surviennent en première semaine de vie avec le risque le plus élevé au premier jour de vie [1] et les principales causes de cette mortalité sont la prématurité et l'infection néonatale [2–5]. Cependant, pour réduire la mortalité néonatale, il s'avère aussi important de considérer les facteurs associés à la morbi-mortalité néonatale. C'est ainsi que nous nous sommes intéressés au transfert des nouveau-nés dans l′unité de soins néonataux car il représente un indicateur de morbidité qui peut être utilisée pour concevoir et mettre en œuvre des interventions permettant d'améliorer la santé et augmenter la survie néonatale.

Le transfert d'un nouveau-né nécessite une organisation rigoureuse et des compétences spécifiques du personnel médical et paramédical afin d'anticiper et de prévenir au mieux les incidents ou accidents qui pourraient survenir au cours du transfert. En France, l′organisation des soins en périnatalité, sur la base d′un réseau régional et de la mise en place de structures dédiées à l′orientation des mères et des enfants depuis quelques années, a permis de réduire très significativement le nombre de transferts postnataux de grands prématurés [6, 7]. L’état actuel de la ville de Lubumbashi et de ses environs voire de la province du Katanga est marqué par l'indisponibilité des unités néonatales spécialisées dans la plupart des centres médicaux où se produisent les accouchements. Certains d′entre eux n′ont même pas les ressources pour le transport néonatal vers des hôpitaux de niveau tertiaire. C'est ainsi que cette étude s'est fixée comme objectifs de déterminer la fréquence du transfert néonatal extrahospitalier à l'unité de néonatologie et d'identifier les facteurs de risque de mortalité dans notre milieu.

## Méthodes

L’étude est prospective et analytique menée sur une période de douze mois allant du 1er janvier 2012 au 31 décembre 2012. L’étude cible tous les nouveau-nés transférés des maternités extérieurs, traités et suivis dans le service de néonatologie de l'hôpital Sendwe pendant la période susmentionnée. A été retenu dans notre étude, tout nouveau-né (âgé d'au plus 7 jours) transféré d'une maternité hors de Sendwe vers le service de néonatologie de l'hôpital Sendwe et ayant été prise en charge jusqu’à sa sortie quel que soit l'issue (guérison ou décès). Tout nouveau-né transféré mais dont la sortie n'a pas été autorisée par un membre du staff médical était exclu ainsi que tout nouveau-né issu d'une grossesse multiple.

La sélection des cas a été faite de façon systématique (exhaustive). Au total, 183 cas de transfert néonatal extrahospitalier ont été enregistré parmi lesquels 150 nouveau-nés ont retenus sur base de nos critères de sélection ci-dessus ont fait l'objet d'analyse. Les caractéristiques maternelles (âge, parité, état-civil, profession, niveau d’étude, antécédents médicaux et toxicologiques), perpartales (âge gestationnel, état de la poche des eaux, voie d'accouchement) et néonatales (âge lors du transfert, sexe, poids de naissance, moyen de transfert, motif de transfert et évolution (guérison ou décès)) ont été étudiés. La saisie et l'exploitation des données ont été faites sur le logiciel Epi Info 2011 (version 70.8.3). L'analyse et l'interprétation ont utilisé le calcul de la proportion, de la moyenne et l’écart-type, le test t de Student, le test de Khi carré (ou celui de Fischer lorsque recommandé), l'intervalle de confiance à 95% et le seuil de signification de 5%.

## Résultats

### Fréquence

Au cours de notre étude qui a couvert la période allant du 1er janvier au 31 décembre 2012 soit une année, le service de néonatologie de l'hôpital Sendwe a enregistré 1412 admissions dont 183 cas de transfert extrahospitalier, soit une fréquence 12,9%.

### Caractéristiques maternelles (Tableau 1)

L’âge maternel moyen est de 24,9±5,8 ans allant de 15 à 41 ans dans le groupe de nouveau-nés décédés alors qu'il est de 25,7±6,9 ans allant de 15 à 42 ans dans le groupe de nouveau-nés guéris. La comparaison de ces deux moyennes ne donne pas de différence statistiquement significative (t = 0,62; p = 0,5300). La proportion des nouveau-nés décédés était de 34,3% chez les mères âgées de moins de 20 ans contre 25,2% chez les mères de plus de 20 ans; l'analyse statistique ne montre pas de différence significative en comparant ces deux proportions (p = 0,4023). La parité moyenne est de 3,3±2,0 allant de 1 à 9 dans le groupe des nouveau-nés décédés alors qu'elle était de 3,2±2,5 allant de 1 à 12 dans le groupe de nouveau-nés guéris; aucune différence statistique significative n'a été trouvé en comparant les deux moyennes (t = 0,05; p = 0,9573). En comparant les proportions des nouveau-nés décédés chez les mères ayant une parité

**Tableau 1:** Mortalité néonatale et caractéristiques maternelles

Paramètre	Décès (n = 41)		Guérison (n = 109)		P	OR (ICà95%)
	n	%	n	%		
Age maternel						
<20 ans	12	34,3	23	65,7	0,4023	1,54 (0,68-3,49)
≥20 ans	29	25,2	86	74,8	-	1
*Moyenne*	*24,9±5,8 ans*		*25,7±6,9 ans*		*0,5300*	
Parité						
<5	31	28,4	78	71,6	0,7714	1,23 (0,53-2,81)
≥5	10	24,4	31	75,6	-	1
*Moyenne*	*3,3±2,0*		*3,3±2,5*		*0,9573*	
Etat-civil						
Mariées	38	27,5	100	72,5	0,8818	1,14 (0,29-4,43)
Singletons^a^	3	25	9	75	-	1
Profession						
Occupées^b^	5	71,4	2	28,6	0,0246*	7,43 (1,38-39,97)
Ménagères	36	25,2	107	74,8	-	1
Niveau d'instruction						
Analphabète	1	25	3	75	0,6336	1,17 (0,11-11,82)
Primaire	11	35,5	20	64,5	0,2043	1,93 (0,81-4,62)
Secondaire	23	22,1	81	77,9	-	1
Supérieur	6	54,5	5	45,5	0,0465*	4,22 (1,18-15,10)
Antécédents toxicologiques^c^						
Présents	7	46,7	8	53,3	0,1427	2,59 (0,87-7,70)
Absents	34	25,2	101	74,8	-	1
Antécédents médicaux^d^						
Présents	27	28,7	67	71,3	0,7599	1,20 (0,56-2,56)
Absents	14	25	42	75	-	1

n: effectif

%: pourcentage

* Test statistique significatif

OR: odds ratio

ICà95%: intervalle de confiance à 95%

a Célibataires, veuves, divorcées

b Travailleuses, vendeuses

c Tabac, alcool

d Hypertension artérielle, diabète, malnutrition

Quant à l’état-civil, la proportion des nouveau-nés décédés chez les mariées n'est pas statistiquement différente de celle retrouvée chez les mères singletons (p = 0,8818). En ce qui concerne la profession, nous constatons que les nouveau-nés des mères occupées (c'est-à-dire celles qui avaient une occupation) mourraient plus que ceux des ménagères (p = 0,0246) et avaient un risque de décès de 7 fois (OR = 7,43 (1,38-39,97)). Concernant le niveau d'instruction, nous remarquons que les nouveau-nés des mères de niveau supérieur décédaient plus que ceux des mères des niveaux inférieurs (analphabètes, primaires et secondaires) (p = 0,0465) et avaient un risque de 4 fois (OR= 4,22 (1,18-15,10)). Bien que les proportions des nouveau-nés décédés chez les mères ayant des antécédents médicaux et toxicologiques soient élevées, l'analyse statistique ne montre aucune différence avec celles retrouvées chez les mères sans antécédent (p > 0,05).

### Caractéristiques perpartales (Tableau 2)

L’âge gestationnel moyen à la naissance est de 33,4±4,5 SA allant de 25 à 41 SA dans le groupe de nouveau-nés décédés alors qu'il est de 36,9±3,1 SA allant de 31 à 42 SA dans le groupe de nouveau-nés guéris. La comparaison de ces deux moyennes ne donne pas de différence statistiquement significative (t = 5,53; p = 0,0000). La proportion des nouveau-nés décédés était de 37,5% avec un âge gestationnel 0,05).

**Tableau 2:** Mortalité néonatale et caractéristiques perpartales

Paramètre	Décès (n = 41)		Guérison (n = 109)		P	OR (ICà95%)
	n	%	n	%		
**Age gestationnel**						
<37 SA	30	37,5	50	62,5	0,0050*	3,21 (1,46-7,06)
≥37 SA	11	15,7	59	84,3	**-**	1
*Moyenne*	*33,4±4,5 SA*		*36,9±3,1 SA*		0,0000*	
**Etat des membranes**						
Rompues	32	28,1	82	71,9	0,8840	1,17 (0,49-2,76)
Intactes	9	25	27	75	-	1
**Aspect duliquide amniotique**						
Clair	29	26,4	81	73,6	-	1
Jaunâtre	2	28,6	5	71,4	0,7540	1,11 (0,20-6,07)
Méconial	10	30,3	23	69,7	0,8236	1,21 (0,51-2,85)
**Mode d'accouchement**						
VB dystocique	8	50,0	8	50,0	0,0125*	6,50 (1,54-27,38)
VB eutocique	29	27,9	75	72,1	0,1482	2,51 (0,80-7,83)
Césarienne	4	13,3	26	86,7	-	1

n: effectif

%: pourcentage

* Test statistique significatif

OR: odds ratio

ICà95%: intervalle de confiance à 95%

VB: voie basse

### Paramètres néonataux (Tableau 3)

S'agissant du sexe de nouveau-nés, nous constatons que 33,7% des nouveaux-nés de sexe masculin sont décédés contre 17,2% de sexe féminin; la comparaison de ces deux fréquences montre une différence statistiquement significative (p = 0,0440) et ceux-ci présentent un risque de décès de 2 fois (OR = 2,43 (1,08-5,46)). En ce qui concerne le poids de naissance, nous constatons que la moyenne était de 1819±819 grammes variant entre 770 et 3510 grammes chez les nouveau-nés décédés alors qu'elle est de 2353±632 grammes variant entre 1000 et 3800 grammes; la comparaison de ces deux moyennes donne une différence statistiquement significative (t = 4,24; p = 0,0000). Nous constatons que le poids de naissance

**Tableau 3:** Mortalité néonatale et caractéristiques néonataux

Paramètre	Décès (n = 41)		Guérison (n = 109)		P	OR (ICà95%)
	n	%	n	%		
Sexe						
Féminin	10	17,2	48	82,8	-	1
Masculin	31	33,7	61	66,3	0,0440*	2,43 (1,08-5,46)
Poids de naissance						
<1500 grammes	21	75,0	7	25,0	0,0000*	15,3 (5,73-40,78)
≥1500 grammes	20	16,4	102	83,6	-	1
*Moyenne*	*1818,9±817,9 grammes*		*2352,6±631,5 grammes*		0,0000*	
Notion de réanimation						
Non	12	23,5	39	76,5	-	1
Oui	29	29,3	70	70,7	0,5775	1,34 (0,61-2,93)
Motif de transfert						
Autres**	3	12	22	88	-	1
Infection	16	21,6	58	78,4	0,3858	2,02 (0,53-7,62)
Prématurité	22	43,1	29	56,9	0,0087*	5,56 (1,47-20,98)
Moyen de transport						
Pied	28	25,5	82	74,5	-	1
Taxi	13	32,5	27	67,5	0,5163	1,41 (0,64-3,10)

n: effectif

%: pourcentage

* Test statistique significatif

OR: odds ratio

ICà95%: intervalle de confiance à 95%

** Détresse respiratoire, traumatisme

## Discussion

### Fréquence

Au cours de la période d’étude, le service de néonatologie de l'hôpital Sendwe a enregistré 1412 admissions dont 183 cas de transfert extrahospitalier soit une fréquence 12,9%. Ces nouveau-nés venaient des maternités ou des services de néonatologie d'autres hôpitaux vers le service de néonatologie de l'hôpital Sendwe, qui est un hôpital de niveau III. Dans une série de 760 transferts de nouveau-nés au CHU de Gabriel Touré de Bamako, 86,7% (659 nouveau-nés) venaient d'une autre structure sanitaire [8]. Dans son étude portant sur la morbidité et mortalité néonatales de 2002 à 2006 au CHU pédiatrique Charles de Gaulle de Ouagadougou (Burkina Faso), Kouéta a enregistré un taux de transfert extrahospitalier de 70% [9]. Le taux de transfert extrahospitalier faible dans notre étude serait dû au fait que dans notre milieu la majorité des hôpitaux ne visent que l'aspect économique et de ce fait ne transferent pas directement les nouveau-nés à problème dans les services spécialisés. Et lorsqu'ils le font, c'est après plusieurs heures; c'est ainsi que nous avons constaté que 43,3% des nouveau-nés dans notre étude ont été transférés après plus de 24 heures suivant leur naissance.

### Caractéristiques maternelles

L’âge maternel moyen est de 25,5±6,6 ans avec près de 52% des mères âgées de moins de 25 ans. La moyenne de la parité est de 3,3±2,4; elles étaient primipares dans 28,7% ou grandes multipares dans 27,4%. La majorité des mères (92%) étaient mariées et 95,3% des mères étaient des ménagères (ou femmes au foyer). Près de 70% des mères avaient un niveau d'instruction secondaire. Hoan, dans une étude réalisée à Hanoï au Vietnam, rapporte un âge maternel moyen de 28,2±5,4ans et 45% de femmes au foyer [5]. Pour Nagalo, cette moyenne d’âge est de 28,3±5,9 ans et 43,9% sont des primipares [10]. Ces différences seraient probablement expliquées par la mixité des populations étudiées par les auteurs précédemment cités, c'est-à-dire que les mères étudiées ne venaient pas seulement du milieu urbain, comme dans notre étude, mais aussi du milieu rural.

La recherche des paramètres maternels ayant influencés la mortalité néonatale a montré que le fait d'avoir une occupation et le niveau d’étude supérieur ont eu un impact sur la mortalité. Nos résultats divergent de ceux de Dicko-Traoré qui lui rapporte un faible niveau d’étude [8]. Ceci s'expliquerait par le fait que le niveau d'instruction est souvent corrélée à la profession; c'est-à-dire qu'une femme travailleuse a un certain niveau d’étude. C'est ainsi qu'il a été démontré dans plusieurs études menées dans le monde qu'une femme qui travaille (donc celle qui a une occupation) est soumise à un certain nombre de risques professionnels: stress, travail physique intense, contraintes organisationnelles, produits chimiques, agents biologiques, rayonnement; l'implication de ces risques professionnels surtout du stress (physique ou psychologique) dans la survenue de faible poids en général et dans la naissance prématurée en particulier a été largement débattue dans plusieurs études [11–13].

L’âge maternel, la parité et l’état-civil n'ont influencé en aucun cas la mortalité néonatale dans notre étude. Les facteurs maternels de risque de décès néonatal rapportés diffèrent selon les études; c'est ainsi que chez Nagalo, ce sont la primigestité et le faible nombre de CPN [10]. Dans l’étude de Bobossi, c'est le jeune âge qui a été mis en évidence [4] et chez Azoumah, c'est la primiparité [3]. Dans une revue de la littérature faite par Lawn sur les facteurs de risque associés au décès néonatal ou périnatal, l’âge maternel (35 ans), la primigestité, la parité >6 et les mauvais antécédents obstétricaux ont été mentionnés comme étant de paramètres maternels très significativement liés à la mortalité des nouveau-nés [14].

### Paramètres périnataux

#### Mode d'accouchement

Les nouveau-nés issus par voie basse dystocique décédaient 7 fois que ceux nés par voie basse eutocique ou voie haute (césarienne) et la moitié d'entre eux sont décédés. Dans notre étude, la voie basse était la voie prépondérante d′accouchement mais la dystocie survenant au cours de cette voie a constitué un facteur de risque de décès néonatal. Cette voie basse dystocique est source d'asphyxie à la naissance et donc de décès si des soins adéquats de réanimation néonatale ne sont pas appliqués; ce constat est identique à celui de Lawn [14]. Pour prévenir l′asphyxie néonatale, des soins prénataux de qualité permettant de dépister les situations à risque et une assistance à l′accouchement par du personnel qualifié sont fondamentaux. Nagalo, dans son étude, souligne que la césarienne semblait être un facteur protecteur de décès néonatal et trouve que le risque qu'un nouveau-né était de près de 3 fois lorsqu'il était né par voie basse (OR = 2,64 (1,55-4,50); p = 0,000) [10].

#### Poids de naissance

Notre étude rapporte que le très faible poids de naissance (<1500 grammes) est indicé d’un risque de décès de 15 fois. En effet la mortalité sextuple lorsque le poids de naissance est faible, chez les moins de 1500 g elle est de 75% contre 16,4% chez les plus de 1500 g. Il ressort que le poids de naissance est un facteur prédictif significatif de la mortalité néonatale. Ceci a été rapporté par d´autres auteurs des pays en développement [4, 5, 8–10, 15]. Les très faibles poids de naissance dans notre milieu causent des sérieux problèmes au personnel de santé du point de vue thérapeutique dans le cadre où l'insuffisance en équipement est à décrier dans la majorité d'institutions hospitalières quel que soit le niveau que peut occuper cette institution. Aux Etats-Unis, au Japon et en Corée du Sud, le taux de survie chez les très faible poids de naissance est respectivement de 92,6% (en 2006), 92,0% (en 2009) et 85,7% (en 2011) [16]. Ces résultats ne sont que le reflet de l'organisation et de l'avancé considérable notées dans le domaine de la périnatologie.

#### Age gestationnel

La proportion des décédés était plus élevée chez les nouveau-nés dont l’âge gestationnel était <37 SA que chez ceux à âge gestationnel ≤37 SA avec un risque de 3 fois (p<0,05). L’âge gestationnel parait être un bon prédicteur de la mortalité néonatale dans notre étude. Ceci est en accord avec les résultats de plusieurs auteurs [5, 9, 10]. Lawn estime que 28% de l'ensemble de décès néonatals sont dus la naissance avant terme [14]. Par contre, pour Onwuanaku, bien que l’âge gestationnel ne soit pas significativement lié à la mortalité néonatale, celui-ci rapporte que cette dernière a tendance à se produire plus à l′âge gestationnel inférieur à 37 semaines [15]. Dans notre milieu, comme dans la plupart des pays en développement qui sont caractérisés par la paupérisation et le sous-équipement des structures sanitaires, l’élevage du prématuré pose des sérieux problèmes et la naissance avant le terme est greffé d'un fort taux de décès néonatal. Le taux élevé de létalité liée à la naissance avant terme retrouvé dans notre étude s'expliquerait par le fait que de la naissance à l'admission dans notre service passant par le transfert, le prématuré ne bénéficie d'aucune prise en charge continue comme il le mérite sachant qu'il présente une immaturité physiologique de tous les organes (immaturité pulmonaire, faibles réserves glucidiques, immaturité de la thermorégulation, immaturité immunologique, immaturité neurologique).

#### Motif de transfert

Concernant le motif de transfert, nous remarquons que la prématurité était greffée d'un risque de décès de près de 6 fois comparativement aux autres motifs. Ceci s'expliquerait par le fait que dans notre milieu, la majorité de maternités périphériques n'ont pas de moyens de transport spécialisé pour le transfert de nouveau-nés; d'où les nouveau-nés sont transférés dans les mains de parents soit par transport en commun soit à pied les exposant ainsi à l'hypothermie et l'hypoglycémie car il ne bénéficie ni de couvertures thermiques ni d'aucun traitement tout le long du trajet vers notre service. La prématurité et ses complications font partie de trois principales causes de mortalité néonatale dans les pays en développement et occupent la première position dans plusieurs études notamment celles de Ngoc-Nguyen (menées dans 6 pays) et de Cissé (menée au Sénégal) [17, 18]. Ainsi la prévention de l'accouchement avant terme est nécessaire pour obtenir une réduction sensible de la mortalité néonatale précoce. Les deux autres sont les infections néonatales et la détresse respiratoire (asphyxie néonatale) [10, 14, 19–21]. Selon Lawn, ces trois pathologies, à elles seules, sont à l'origine de 86% des décès néonatals dans le monde [14]. Mais ce constat n'est pas le même pour Kouéta qui lui trouve que la mortalité néonatales de 2002 à 2006 au CHU pédiatrique Charles de Gaulle de Ouagadougou (Burkina Faso) était due aux infections néonatales dans 81,3% des cas suivies des malformations congénitales et les intoxications aiguës accidentelles [9]. En vue de la prise en charge optimale de la mère et de l'enfant, l'accouchement de certaines parturientes à risque nécessite des connaissances, des capacités et un équipement spécialisés que l'on trouve dans les institutions hospitalières de niveau III. C'est la raison pour laquelle en 2012, la Société Suisse de Néonatologie avait recommandé de transférer les parturientes à risque avant l'accouchement prévu ou imminent dans un centre de périnatologie équipé de soins intensifs néonataux [22].

#### Moyen de transfert

S'agissant du moyen de transport utilisé, le transport public a été utilisé dans 26,7% des cas, dans la majorité des cas, le transport était fait à pieds et en aucun cas l'ambulance n'a été utilisée. Le transfert d'un nouveau-né nécessite une organisation rigoureuse et des compétences spécifiques du personnel médical et paramédical afin d'anticiper et de prévenir au mieux les incidents ou accidents qui pourraient survenir au cours du transfert. Au niveau de la ville, le transfert médicalisé du nouveau-né n'est nullement pratiqué en raison du manque d'ambulances adaptées au nouveau-né, de l'insuffisance des moyens financiers. Suite à la non médicalisation du moyen de transport de nouveau-nés, nous constatons que les proportions des nouveau-nés décédés ne diffèrent pas statistiquement quel que soit le moyen de transport utilisé. Les nouveau-nés sont amenés enveloppés et transportés entre les mains d'un membre de la famille et rarement accompagnés d'un personnel de santé qualifié et dans aucun cas le service a été informé à l'avance du transfert. Ce constat est semblable à ceux faits par d'autres auteurs ayant travaillé dans les pays en développement [8, 23]. Verónica, dans une étude menée à Jalico (Méxique) dont le but était d′évaluer l′impact du transfert médicalisé sur la morbi-mortalité néonatale, rapporte une nette amélioration du taux de morbidité et une baisse de la mortalité lorsqu'il compare la période d'avant la mise en place du programme de transport médicalisé et celle où le transport était médicalisé [24].

#### Sexe

L’étude montre que le sexe masculin soit significativement lié à la mortalité néonatale (p < 0,05) et comportant 2 fois le risque de décès que le sexe opposé. Plusieurs études rapportent que les nouveau-nés de sexe masculin ont un taux de mortalité plus élevé que leurs homologues féminins [3, 4, 8, 15, 19, 25, 26] mais aucune explication suffisante ne justifie encore ce phénomène. Cette surmortalité masculine peut être en raison de certains facteurs tels que les facteurs génétiques et selon l'Organisation Mondiale de la Santé, pour ce qui est de la survie durant la période néonatale, les filles auraient un avantage biologique [14, 20, 27].

## Conclusion

Le bilan de la mortalité néonatale est lourd dans les pays en développement où les nouveau-nés continuent de mourir pour des causes souvent évitables. La réduction de la morbidité et la mortalité néonatales passe par une amélioration du système de référence des nouveau-nés dans notre milieu.

## References

[1] Zupan J (2005). Perinatal Mortality in Developing Countries. N Eng J Med..

[2] Bezzaoucha A, El Kebboub A, Aliche A (2010). Évolution de la mortalité néonatale au CHU de Blida (Algérie) de 1999 à 2006. Bull Soc Pathol Exot..

[3] Azoumah KD, Balaka B, Aboubakari AS, Matey K, Yolou A, Agbere AD (2010). Morbidité et mortalité néonatales au CHU Kara (Togo). Med Afr Noire..

[4] Bobossi-Serengbe G, Sana Deyamissi TS, Diemer HC, Gaudueille A, Gresenguet G, Mandaba JL, Siopathis RM (2004). Morbidité et mortalité néonatales au Complexe pédiatrique de Bangui (Centrafrique). Med Afr Noire..

[5] Hoan TP, Bao TV, Phong DN, Huong NT, Manirankunda L, Boelaert M (2000). Mortalité néonatale précoce à l'Hôpital de gynécologie-obstétrique de Hanoï, Vietnam. Bull Soc Pathol Exot..

[6] Verónica RM, Gallo LL, Medina DR, De la Torre MG, Mancilla J-L, Amezcua MM (2011). Safe neonatal transport in the state of jalisco: impact of the STABLE program on morbidity and mortality. Bol Med Hosp infantile Mex..

[7] Ancel PY, du Mazaubrun C, Bréart G (2001). Grossesses multiples, lieu de naissance et mortalité des grands prématurés: premiers résultats d'EPIPAGE Ile de France. J Gynecol Obstet Biol Reprod..

[8] Dicko-Traoré F, Sylla M, Diakité AA, Soilihi A, N'Diaye MD, Togo B, Diakité FL, Konaté D, Traoré B, Sidibé T, Keïta MM (2010). Problématique du transfert néonatal vers le service de pédiatrie du CHU Gabriel Touré de Bamako. Mali médical..

[9] Kouéta F, Yé D, Dao L, Néboua D, Sawadogo A (2007). Morbidité et mortalité néonatales de 2002 à 2006 au CHU pédiatrique Charles de Gaulle de Ouagadougou (Burkina Faso). Cahiers Santé..

[10] Nagalo K, Dao F, Tall FH, Yé D (2013). Morbidité et mortalité des nouveau-nés hospitalisés sur 10 années à la Clinique El Fateh-Suka (Ouagadougou, Burkina Faso). Pan African Medical Journal..

[11] Dole N, Savitz DA, Hertz-Picciotto I, Siega-Riz AM, McMahon MJ, Buekens P (2002). Maternal Stress and Preterm Birth. Oxford Journals Medicine American Journal of Epidemiology..

[12] Vendittelli F, Lachcar P (2002). Menace d'accouchement prématuré, stress, soutien psychosocial et psychothérapie: revue de la littérature. Gynécologie Obstétrique & Fertilité..

[13] Epiney M, Boulvain M, Irion O (2011). Facteurs de risque psychosociaux et accouchement avant terme. Rev Med Suisse..

[14] Lawn JE, Cousens S, Zupan J (2005). 4 millions neonatal deaths: When? Where? Why?. Lancet..

[15] Onwuanaku CA, Okolo SN, Ige KO, Okpe SE, Toma BO (2011). The effects of birth weight and gender on neonatal mortality in north central Nigeria. BMC Research Notes..

[16] Hahn W-H, Chang J-Y, Chang YS, Shim KS, Bae CW (2011). Recent Trends in Neonatal Mortality in Very Low Birth Weight Korean Infants: In Comparison with Japan and the USA. J Korean Med Sci..

[17] Ngoc Nguyen NT, Merialdi M, Abdel-Aleem H (2006). Causes of stillbirths and early neonatal deaths: data from 7393 pregnancies in six developing countries. Bull WHO..

[18] Cissé CT, Yacoubou Y, Ndiaye O, Diop-Mbengue R, Moreau JC (2006). Evolution de la mortalité néonatale précoce entre 1994 et 2003 au CHU de Dakar. J Gynecol Obstet Biol Reprod..

[19] Akaffou E, Amon Tanoh-Dick F, Lasme-Guillao BE, Yenan JP (2011). Mortalité néonatale et niveaux de diagnostic au Centre Hospitalier Universitaire de Yopougon (Abidjan). Mali Médical..

[20] Organisation mondiale de la santé (2005). La survie du nouveau-né. The Lancet.

[21] Mmbaga BT, Lie RT, Olomi R, Mahande MJ, Kvåle G, Daltveit AK (2012). Cause-specific neonatal mortality in a neonatal care unit in Northern Tanzania: a registry based cohort study. BMC Pediatrics..

[22] Société Suisse de Néonatologie (2012). Prise en charge et réanimation du nouveau-né: recommandations révisées de la Société Suisse de Néonatologie. Paediatrica.

[23] Mohammad K, Seyed-Hossein F, Farzaneh Z (2004). Neonatal transport in Tehran: a cause for much concern. Arch Iranian Med..

[24] Verónica RM, Gallo LL, Medina DR, de la Torre MG, Mancilla JLS, Amezcua MM, Huizar LMA, Padilla ER, Hernández HAG, de Padilla JAG (2011). Safe neonatal transport in the state of Jalisco: impact of the STABLE program on morbidity and mortality. Bol Med Hosp infantile Mex..

[25] Vierin N'Zamey Y, Maladjou Kondjo J, Gahouma D, Imboua L, Mongi P, Moussavou A Enquete sur la mortalite neonatale au Gabon.

[26] Cissé CT, Martin SL, Ngoma SJ, Mendes V, Diadhiou F (1996). Mortalité néonatale précoce à la maternité du CHU de Dakar: situation actuelle et tendances évolutives entre 1987 et 1994. Med Afr Noire..

[27] Kane F, Edward MC (2006). Gender imbalance in infant mortality: a crosssectional study of social structure and female infanticide. Soc Sci Med..

